# Death of Dr. Paul F. Eve

**Published:** 1877-11-20

**Authors:** 


DEATH OF DR. PAUL F. EVE.—This good man and eminent Surgeon died suddenly in Nashville, on the 3d inst. He was, says the Chattanooga *Dispatch*, by the bed-side of a patient when he suddenly felt a pain in his wrist. He sat down in a chair and dropped his head in his hands. He was laid upon a couch and expired in a few moments without a struggle. He complained of the pain in his wrist at 6: 15 a. m. and by 6: 25 he was dead. Doubtless he died of an affection of the heart, the symptoms of which he had long felt.

"Dr. Eve stood at the head of his profession as a skillful surgeon. As an operator and author he had gained an enviable reputation. He will be painfully missed by his associates and friends in Nashville, and various other cities where he has been called from time to time, by the demands of suffering humanity. He was a bold operator and experienced physician; a true friend and a noble citizen."

As we recur to the period long years ago when, in the prime and vigor of his life, he lectured in Augusta, to large and intelligent classes, we are impressed with both pleasant and melancholy reminiscences. The kind treatment and cordial greetings which, individually, we often received at his hands, we can never forget; and long will hundreds of his surviving pupils cherish the generous and cordial courtesies which he ever extended to the members of his class.

It is sad to know that his manly form is cold in death, and that the eloquence of his voice is forever hushed! Truly the spirit of a great and good man has fled forever, and the Profession has lost one of her noblest and most talented members. Peace to his memory! Peace to his ashes!

